# Internal Secretion and the Ductless Glands

**Published:** 1912-12

**Authors:** 


**Internal Secretion and the Ductless Glands.** By SWALE VINCENT, M.D. Lond., D.Sc. Edin. With a Preface by Professor E. A. SCHAFER, F.R.S. Pp. 463. London: Edward Arnold. 1912. Price 12s. 6d.

This book in many respects supplies a real need. The amount of experimental work done in recent years on the ductless glands is enormous, and is brought forward at such a rate that it is almost impossible to keep up with it, especially as it is scattered through the journals of all nations. At the same time, the practitioner of medicine knows that the subject is one of pressing and increasing importance to him in his daily work, and one with which he must be thoroughly conversant if he is to do justice to his patients. He will therefore welcome this work from such an authority on the subject as Professor Swale Vincent, who himself has contributed so largely to our knowledge of the action of these glands. The book will be valuable as a reference handbook to the literature of this branch of medicine.

The introductory chapters on the definition and nature of internal secretions, and of hormones, and the critical account of the methods of investigation and the results obtained from them, are clearly written, and of especial usefulness to those not familiar with the subject. In the remainder and chief bulk of the book each of the ductless glands has a separate chapter devoted to it, in which we note for especial mention the sections on the development and comparative anatomy of each organ, of their physiological activities and applications in the treatment of disease. The author is commendably careful in not rushing to hasty conclusions on insufficient data, and throughout prefers to leave a question in suspense where the facts so far obtained do not warrant a decisive opinion. He is thus a trustworthy guide. Although the amount of knowledge gained in the last few years as to the action of these glands is remarkable and, as Professor Schäfer observes in his Introduction, surpasses that of organs whose physiological action has been long known, yet one rises from the perusal of the book with a sense of how much more there is still to be learnt. Especially is this the case, perhaps, as regards the very interesting and important subject of the interaction of these glands upon one another in the normal economy of the body.

A useful summary of the positive knowledge so far gained concludes the section on each gland. We have only one or two small criticisms to make, and these are that those summaries might be still further extended, as the reader will be sometimes puzzled in selecting the grain from the chaff in the multitude of experiments, often with conflicting results, which are here presented to his notice ; that there are a good many verbal errors, and that the writing sometimes shows marks of haste. In a work involving so much labour, these are minor defects and we cordially commend the book to our readers as one calling for careful perusal, and in which is collected together a mass of information not otherwise accessible. The illustrations are good, copious, and mostly original ; they admirably elucidate the text. The extensive bibliography at the end of the book is well worth the trouble it must have involved, and there is that indispensable part of a work of this kind—a good index.

